# Cytokine response in human leptospirosis with different clinical outcomes: a systematic review

**DOI:** 10.1186/s12879-020-04986-9

**Published:** 2020-04-07

**Authors:** Indika Senavirathna, Devarajan Rathish, Suneth Agampodi

**Affiliations:** 1 Department of Biochemistry, Faculty of Medicine and Allied Sciences, Rajarata University of Sri Lanka, Saliyapura, Sri Lanka; 2 Department of Pharmacology, Faculty of Medicine and Allied Sciences, Rajarata University of Sri Lanka, Saliyapura, Sri Lanka; 3 Department of Community Medicine, Faculty of Medicine and Allied Sciences, Rajarata University of Sri Lanka, Saliyapura, Sri Lanka

**Keywords:** Cytokines, Chemokines, MAT assay, ELISA, Severe leptospirosis, Weil’s disease

## Abstract

**Background:**

Leptospirosis is a neglected zoonotic disease which is a major challenge for clinicians and public health professionals in tropical countries. The cytokine storm during the second (immune) phase is thought to be a major contributory factor for the leptospirosis disease severity. We aim to summarize evidence for cytokine response in leptospirosis at different clinical outcomes.

**Methods:**

A systematic review was carried out to examine the cytokine response in leptospirosis patients using relevant scientific databases. Reference lists of the selected articles were also screened. Quality of the selected studies was assessed by using the National Institutes of Health Quality Assessment Tool for Observational Cohort and Cross-Sectional Studies.

**Results:**

Of the 239 articles retrieved in the initial search, 18 studies fulfilled the selection criteria. India and Thailand have produced the highest number of studies (17% each, *n* = 3). The majority were comparative cross-sectional studies (72%, *n* = 13). Overall the quality of the selected studies was fair regardless of few drawbacks such as reporting of sample size and the lack of adjustment for confounders. Microscopic agglutination test (67% - 12/18) and enzyme-linked immunosorbent assay (50% - 9/18) were commonly used for the confirmation of leptospirosis and the measurement of cytokines respectively. IL-1b, IL-2, IL-4, IL-6, IL-8, IL-10 and TNF-α levels were found to be significantly higher in severe than in mild leptospirosis. There were equivocal findings on the association between IL-1β, TNF-α and IL-10/TNF-α ratio and disease severity.

**Conclusions:**

Leptospirosis had a wide-range of elevated cytokines. However, prospective studies in-relation to the onset of the symptom are required to better understand the pathophysiology of cytokine response in leptospirosis.

## Background

Recent outbreaks of leptospirosis in different parts of the world show that the disease is re-emerging [1, 2]. The clinical picture of the disease is diverse and vary from flu-like illness to severe disease with multi-organ failure and death [3]. Clinical management of leptospirosis is always a challenge to the physicians due to the highly variable nature of its presentation. Traditionally, complications of leptospirosis are described in the second (immune) phase of the disease [3]. The most common reported causes of death in leptospirosis included respiratory, cardiac, or renal failure, and septic shock [4]. Rarely, early complications and death were attributed to the Jarisch-Herxheimer reaction [5].

The variation of disease severity is attributed to the differences in virulence factors of *Leptospira* [6]*,* inoculum size [7] and the host immune response [8], especially via cytokine production. The pathogenesis of leptospirosis is still not fully described; however; the innate immune-mediated complications are described. Several molecules of the infectious agents like lipopolysaccharides, hemolysins, outer membrane protein and the glycolipoprotein have been implicated with the disease [3, 9]. It is proposed that the pathogenesis of leptospirosis is driven through the cytokine response [10].

Many viral infections show an abnormal immune response and cytokine production [11]. Dengue hemorrhagic fever (DHF) is closely linked to the host innate and adaptive immune response. IL-6, IL-8, IL-10, IP-10, MCP-1, TNF-α and vascular endothelial growth factors (VEGF-A) promote clinical manifestations of DHF [12]. Pathogenesis of Ebola virus is influenced by multiple factors out of which excessive inflammatory response is a significant feature [13]. The potential source of inflammatory cytokines during multiple viral hemorrhagic fever infections could vary. Dendritic cells secrete minimal amounts of cytokines since they are targeted by multiple viruses (arenaviruses, bunyaviruses, filoviruses, and flaviviruses). However, monocytes and macrophages appear to be playing a dominant role in secreting inflammatory cytokines during these infections [11]. Severe systemic clinical manifestations of gram-positive sepsis are also attributed to the non-specific pathogen-associated molecular pattern effect of peptidoglycan and lipoteichoic acid which trigger toll-like receptor 2 and induce inflammation. Similarly, lipopolysaccharides has its inflammatory effect through trigger toll-like receptor 4 [14]. The function of the immune system is to eliminate the microbial pathogen by producing pro-inflammatory agents such as cytokines. However, tissue injury could be possible with excessive production of cytokines [15]. We aim to systematically review the previous evidence on cytokine response in leptospirosis at different clinical outcomes.

## Methods

### Eligibility criteria

Full-text articles which reported cytokine response in human leptospirosis were included in this review. Reports in the non-English language were excluded. Reports were not excluded based on the year of publication or patient population.

### Information sources and search strategy

**Fig. 1:**
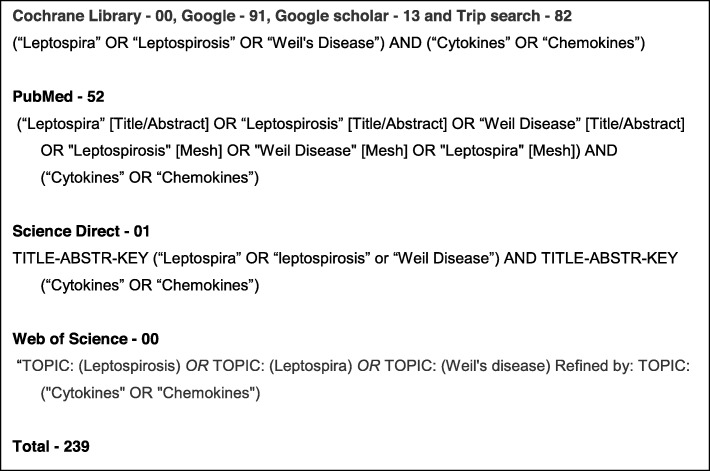
Keywords for databases and the number of search results

The studies published before 31st of August 2018 were identified through systemic searches of electronic databases and cross-checking reference lists of the selected articles. Cochrane-Library, Google-Scholar, Google, PubMed, Science-Direct, Trip and Web of Science were the databases searched using a string of keywords (Fig. 1). Appropriate Medical Subjects Heading (MeSH) and other relevant terms were used to identify published studies.

### Study selection

IS performed a comprehensive literature search. All selected titles and abstracts were independently screened by IS and DR. Full-text articles were assessed when the abstract was unclear. The selected studies were independently reviewed by SA to confirm eligibility.

### Data collection process, data items and data analysis

Year of publication, demographic details, study design, methods of leptospirosis confirmation, types and methods of cytokine analysis and outcomes were extracted from the selected studies. The Population, Intervention, Comparator and Outcome (PICO) were patients with leptospirosis / severe disease / fatal outcome, cytokine analysis, healthy individuals/patients with mild disease/survivors and the cytokine levels respectively. SI units were used for presenting data. The data were analysed using Microsoft Excel (Additional file 1). Descriptive statistics were used to describe the data. The Cochrane guideline for systematic review was followed [16]. The review is presented according to the PRISMA - Preferred Reporting Items for Systematic Reviews guidelines [17]. Quality assessment of the selected studies was done using the National Institutes of Health Quality Assessment Tool for Observational Cohort and Cross-Sectional Studies (Additional file 2) [18]. Graphs were drawn using the GraphPad Prism 8 software [19].

## Results

### Selected studies

**Fig. 2:**
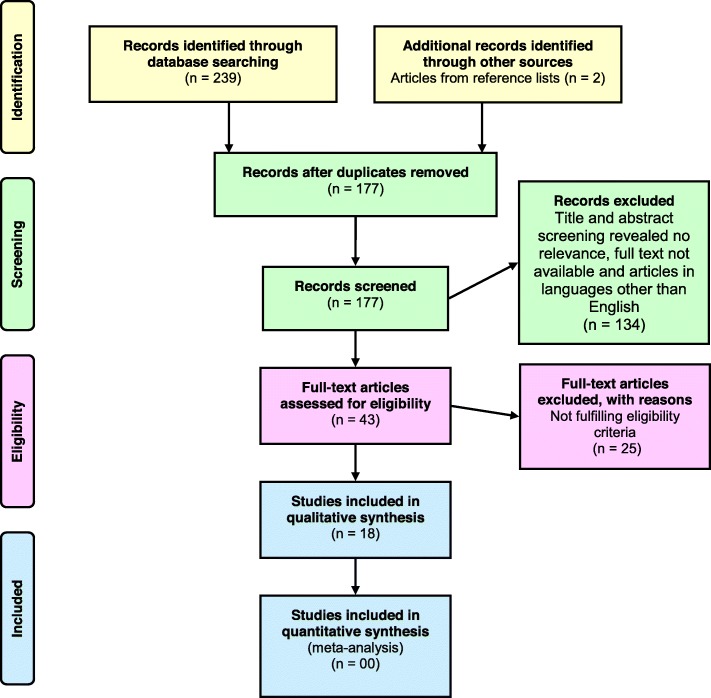
Flow diagram showing the selection process of articles for this review, according to PRISMA 2009

A total of 239 search results were identified via the database search and additionally two articles were identified by screening the reference list (Fig. 2). After removal of duplicates,177 articles were included for the title and abstract screening. Out of the above, 134 articles were excluded due to irrelevance to study objective, unavailability of full text and non-English language. The full-texts of the remaining 43 articles were examined for suitability to the review criteria, and 25 were excluded. After the screening procedure, 18 articles were selected for the systematic review [20–37]. Selected articles were published over 18 years from 2000 to 2018. Quality assessment of the selected studies revealed a lack of reporting of sample size justification (or power description or variance or effect estimates), blinding of outcome assessors to the exposure status and the statistical adjustment of potential confounding variables. Summary of the quality assessment of the selected studies is given in Additional file 2.

### Characteristics of the studies

Most of the studies were conducted in India and Thailand (each 17% - 3/18). The majority of the studies were comparative cross-sectional studies (83% - 15/18). Patients with clinical suspicion (seronegative) (1/18), presumptive diagnosis (2/18), confirmed diagnosis (3/18), exposure (1/18), mild disease (7/18), severe disease (7/18), acute kidney injury (1/18), hepatitis (2/18), scrub typhus (1/18), recovery (2/18) and fatal (1/18) were included as subjects in the selected studies. Healthy controls were used in 11 studies. The timing of the sample collection for cytokine measurement was reported in four studies, and it varied from 3 days to 2.8 years. A total of 1264 subjects participated in the selected studies. Thirteen studies reported the composition of male and female participants and accordingly there were a total of 551 males and 188 females. The mean age of the patients varied from 28.3 to 54.2 years. Microscopic agglutination test (MAT) (67% - 12/18) was commonly used for confirmation of leptospirosis. Other methods were clinical signs, culture, IFA-IgM, IgG, IgM ELISA, Lepto Tek Dri-DOT, Microscopy, nested PCR, PCR and RT-PCR (Additional file 1). Enzyme-linked immunosorbent assay (ELISA) (50% - 9/18) was commonly used for the measurement of cytokines. Other methods were Bio-Plex multiplex cytokine assay (2/18), Bio-PlexTM suspension array system (1/18), Chemiluminescent enzyme immunoassay (1/18), Cytometric beads array multiplex assay (1/18), Flow cytometric analysis (2/18), Flow cytometer (FACSCalibur) inflammatory colorimetric bead array (1/18) and Protein microarray (quantitative RayBIO) (1/18) (Additional file 1).

### Cytokine response in leptospirosis patients

Cytokines Eotaxin, Eotaxin-2, FGF, G-CSF, GMCSF, I-309, ICAM-1, IFN-γ, IL-1β, IL-1α, IL-1RA, IL-2, IL-3, IL-4, IL-5, IL-6, IL-6SR, IL-7, IL-8, IL-9, IL-10, IL-11, IL-12P40, IL-12P70, IL-12, IL-13, IL-15, IL-16, IL-17A, IL-18, IL-21, IL-23, IP-10, MCP-1, MCP-2, MCSF, MIG, MIP-1α, MIP-1β, MIP-1δ, PDGF, PDGF-BB, RANTES, sTNFR1, sTNFR2, TGF-β, TGF-β1, TIMP-2, TNF-α, TNF-β, VEGF were studied in the selected articles (Additional file 1). TNF-α levels were measured in all studies except one.

**Fig. 3:**
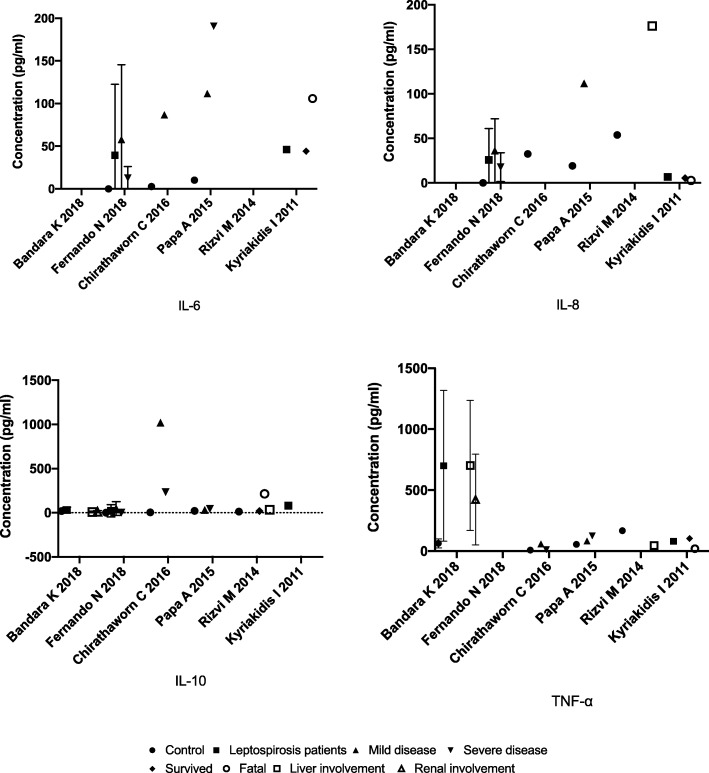
Mean values of IL-6, IL-8, IL-10 and TNF-α reported among leptospirosis patients with different outcomes and the controls (standard deviation shown where available)

Studies reported Eotaxin-2 [22], G-CSF [22], GM-CSF [22, 33], IFN-γ [22], IL-1β [22], IL-2 [35], IL-4 [22], IL-6 [21, 22, 26, 31, 33, 34], IL-7 [22], IL-8 [21, 23, 26, 31, 33, 36], IL-10 [21, 22, 26, 31, 36], IL-11 [22], IL-12P40 [28], IL-13 [22], IL-17 [22], IL-21 [20], IL-23 [20], IP-10 [27, 33], MCP-1 [22, 33], MIG [27], MIP-1δ [22], sTNFR1 [22], TNF-α [20–22, 31, 34, 35] and VEGF [33] as elevated among leptospirosis patients. In addition, studies also reported IL-1β [21, 26], IL-12P70 [21, 26] and TNF-α [21, 23, 26, 36], levels as low or undetectable in leptospirosis patients and healthy controls. Higher levels of IL-8 [36], IL-10 [36] and lower levels of TNF-α [36] were found to be associated with better prognosis. The first, 5 days of infection showed peak levels of IL-1RA, MCP-1, MIP-1β and TNF-α; the second 5 days had peak levels of GM-CSF, IL-1β, IL-6, IL-8, IL-9, IP-10 and MIP-1α; VEGF peaked at 11–15 days [33]. Tuero et al. 2010 revealed a significantly less peripheral blood mononuclear cell production of IL10 in patients than in controls [25]. Kyriakidis et al. 2011 showed that the day of illness is a determinant of cytokine levels and that the age of the patients correlates with sTNFR1 levels [24]. An involvement of Th17 cells in the immunopathogenesis of leptospirosis was proposed by Bandara et al. 2018 because an increase in IL-17A, IL-21 and IL-23 levels were observed [20]. Latha et al. 2015 demonstrated significant levels of TNF-α and IL-6 in sera which were reactive in *Leptospira tarassovi* outer membrane protein IgM ELISA [34]. Wang et al. 2012 revealed that the secretion of pro-inflammatory cytokines is induced by hemolysin of *Leptospira interogans* in human macrophages [22]. Table 1 summarizes mean (SD) of the five most commonly reported cytokines among leptospirosis patients with different outcomes and the controls (Fig. 3).

**Table 1:** Mean (SD) of the five most commonly reported cytokines among leptospirosis patients with different outcomes and the controls

Study No	Year	Type	Control			Leptospirosis patients			Mild disease			Severe disease			Survived			Fatal			Liver involvement			Renal involvement		
			n	Mean (pg/ml)	SD	n	Mean (pg/ml)	SD	n	Mean (pg/ml)	SD	n	Mean (pg/ml)	SD	n	Mean (pg/ml)	SD	n	Mean (pg/ml)	SD	n	Mean (pg/ml)	SD	n	Mean (pg/ml)	SD
Bandara K	2018	IL-10	12	18.6	10.5	57	30.7	33.2	NR	NR	NR	NR	NR	NR	NR	NR	NR	NR	NR	NR	7	7.9	9.4	14	11.4	11.8
		TNF-α	12	63.0	37.2	57	700.0	619.0	NR	NR	NR	NR	NR	NR	NR	NR	NR	NR	NR	NR	7	702.8	532.3	14	422.5	373.1
Fernando N	2018	IL-6	18	0.1	0.2	51	39.4	83.1	26	57.9	87.7	25	12.8	13.5	NR	NR	NR	NR	NR	NR	NR	NR	NR	NR	NR	NR
		IL-8	18	0.1	0.4	51	25.8	35.2	26	36.2	35.7	25	17.7	16.2	NR	NR	NR	NR	NR	NR	NR	NR	NR	NR	NR	NR
		IL-10	18	0.0	0	51	21.7	68.7	26	48.0	78.1	25	0.9	1.8	NR	NR	NR	NR	NR	NR	NR	NR	NR	NR	NR	NR
Chirathaworn C	2016	IL-6	25	2.7	NR	NR	NR	NR	20	86.7	NR	20	849.2	NR	NR	NR	NR	NR	NR	NR	NR	NR	NR	NR	NR	NR
		IL-8	25	32.5	NR	NR	NR	NR	20	1020.0	NR	20	2244.5	NR	NR	NR	NR	NR	NR	NR	NR	NR	NR	NR	NR	NR
		IL-10	25	4.0	NR	NR	NR	NR	20	1020.0	NR	20	234.7	NR	NR	NR	NR	NR	NR	NR	NR	NR	NR	NR	NR	NR
		TNF-α	25	7.9	NR	NR	NR	NR	20	58.7	NR	20	11.0	NR	NR	NR	NR	NR	NR	NR	NR	NR	NR	NR	NR	NR
Papa A	2015	IFN-γ	20	137.1	NR	NR	NR	NR	7	130.2	NR	47	170.4	NR	NR	NR	NR	NR	NR	NR	NR	NR	NR	NR	NR	NR
		IL-6	20	10.2	NR	NR	NR	NR	7	111.6	NR	47	190.8	NR	NR	NR	NR	NR	NR	NR	NR	NR	NR	NR	NR	NR
		IL-8	20	19.2	NR	NR	NR	NR	7	111.6	NR	47	223.6	NR	NR	NR	NR	NR	NR	NR	NR	NR	NR	NR	NR	NR
		IL-10	20	20.4	NR	NR	NR	NR	7	30.2	NR	47	43.4	NR	NR	NR	NR	NR	NR	NR	NR	NR	NR	NR	NR	NR
		TNF-α	20	55.2	NR	NR	NR	NR	7	82.1	NR	47	124.5	NR	NR	NR	NR	NR	NR	NR	NR	NR	NR	NR	NR	NR
Rizvi M	2014	IL-8	30	53.8	NR	NR	NR	NR	NR	NR	NR	NR	NR	NR	NR	NR	NR	NR	NR	NR	46	176.0	NR	NR	NR	NR
		IL-10	30	12.8	NR	NR	NR	NR	NR	NR	NR	NR	NR	NR	NR	NR	NR	NR	NR	NR	46	32.9	NR	NR	NR	NR
		TNF-α	30	167.0	NR	NR	NR	NR	NR	NR	NR	NR	NR	NR	NR	NR	NR	NR	NR	NR	46	46.5	NR	NR	NR	NR
Kyriakidis I	2011	IL-6	NR	NR	NR	44	46.2	NR	NR	NR	NR	NR	NR	NR	39	44.2	NR	5	105.9	NR	NR	NR	NR	NR	NR	NR
		IL-8	NR	NR	NR	44	6.8	NR	NR	NR	NR	NR	NR	NR	39	5.5	NR	5	2.8	NR	NR	NR	NR	NR	NR	NR
		IL-10	NR	NR	NR	44	47.3	NR	NR	NR	NR	NR	NR	NR	39	19.9	NR	5	214.3	NR	NR	NR	NR	NR	NR	NR
		TNF-α	NR	NR	NR	44	79.9	NR	NR	NR	NR	NR	NR	NR	39	103.4	NR	5	19.3	NR	NR	NR	NR	NR	NR	NR

*N* Total, *NR* Not reported, *SD* Standard deviation

### Cytokines response in severe leptospirosis

IL-1β [37], IL-2 [37], IL-4 [37], IL-6 [31], IL-8 [31, 37], IL-10 [31, 37] and TNF-α [35, 37] levels were significantly higher in severe than in the mild group. Elevated levels of IFN-γ [37], IL-6 [26, 37], IL-8 [26, 37], IL-10 [24, 37], sTNFR1 [24] and TNF-α [23] were observed in fatal outcomes. IL-21 and IL-23 were significantly elevated in patients with renal failure than in mild disease [20]. Leptospiral hepatitis showed a suppressed TNF-α and a 3–4 fold increase in liver enzymes along with elevated levels of IL-8 [23]. Liver insufficiency also had elevated levels of IL-23 and TNF-α [20] than in mild disease. A study revealed a significantly lower TNF-α with pulmonary disease [33] while another study had higher TNF-α levels in pulmonary disease [24]. Patients with severe pulmonary haemorrhage had significantly higher levels of IL-5, IL-6, IL-8, and IL-10. IL-6 was associated with severe pulmonary haemorrhage in fatal outcomes even after adjusting for age and duration of symptoms [37].

High IL-10/TNF-α ratio was associated with leptospirosis [36] and fatal outcomes [37]. By contrast, other studies showed that low IL-10/TNF-α ratio was associated with leptospirosis [35], severe disease [32] and fatal outcomes [24]. However, IL-10/TNF-a ratio did not correlate with sex or age [24].

### Cytokines response in leptospirosis treatment

More than five doses of antimicrobial agents (penicillin, ceftriaxone, doxycycline) significantly lowered IL-6 and IL-8 levels, when compared to lower doses. IL-8 levels were negatively associated with the total number of antimicrobial doses administered (penicillin, ceftriaxone, doxycycline) and also with the number of ceftriaxone doses administered [21]. Extended sustained low-efficiency dialysis via hemodiafiltration significantly decreased the post-admission levels of IL-17, IL-7, and MCP-1 compared to the dialysis via hemodialysis among patients with acute kidney injury or acute respiratory distress syndrome following leptospirosis [30].

## Discussion

The review had data from studies which included leptospirosis patients with varying degree of severity. Although, the review produced valuable data on the possible role of cytokines in leptospirosis disease severity, there were equivocal findings with regards to IL-1β, TNF-α and IL-10/TNF- *α* ratio among the selected studies. Failure to measure soluble TNF receptor-1 (sTNFR1) might have led to low levels of TNF-α in some studies, as the bound TNF-α will not be included in the analysis [37]. IL-1β, IL-6 and TNF-α were proposed to be pro-inflammatory cytokines which enhance the inflammatory response in leptospirosis [10]. However, the present review found IL-1β to be low among leptospirosis patients, IL-6 to be elevated and TNF-α to be equivocal. The above findings could be due to the variation in the time of sample collection in relation to the onset of the symptoms. Nevertheless, results among the complicated leptospirosis showed elevated levels of IL-6 and TNF-α which shows a higher degree of pro-inflammatory action. In contrast, IL-10 is thought to be the most important anti-inflammatory cytokine which restores the immunological homeostasis in leptospirosis [10]. In addition, IL-4 and IL-13 restrict the inflammatory response [10]. The present review revealed elevated levels of the above-mentioned anti-inflammatory cytokines among leptospirosis patients and patients with complicated disease. The findings suggest that anti-inflammatory response is also expected to increase in complicated leptospirosis. Therefore, a low IL-10 / TNF-α is most probable among patients with complicated disease.

Differential diagnosis for leptospirosis includes DHF, chikungunya, hanta virus and malaria infections. Similar to the present review, IL-6 [38], IL-8 [38], IL-10 [38, 39], IP-10 [38], MCP-1 [38], TNF-α [40, 41] and VEGF [39] were found to be elevated in DHF, a condition common to almost all endemic settings for leptospirosis. IL-6, IL-12, IL-1RA, IP-10, and MCP-1 were also found to be elevated in chikungunya [42–44]. Hanta virus infection, a disease which can mimic pulmonary leptospirosis, had shown elevation of GM-CSF, IL-2, IL-6, IL-10, IL-12P40, IL-17 and VEGF [45]. Fatal outcomes from *Plasmodium falciparum* malaria showed elevated levels of IL-6, IL-10 and TNF-α than in survival which was comparable to the results on leptospirosis [46]. Cerebral malaria was found to have high levels of TNF-α in fatal outcomes [46]. Moreover, Ebola viral infection showed elevation of pro-inflammatory cytokines IL-6 and IL-8 in non-survivors similar to the present review [47, 48]. It is interesting to note that IL-6 was found to be elevated in leptospirosis, DHF, chikungunya, hanta virus, malaria and Ebola infections. However, cytokine response alone cannot be used to differentiate the above conditions.

Both Papa et al. [33] and Kyriakidis et al. [24] showed that the day of illness is a determinant of cytokine levels. The above fact emphasizes the need of cytokine selection in relation to the day of illness, if we are to use the cytokine levels for disease monitoring. The selected studies were heterogeneous, and the methods used for the diagnosis of leptospirosis and measurement of cytokines varied between studies, making it impossible for proper evidence synthesis. Although, the quality assessment revealed few drawbacks such as reporting of sample size and the lack of adjustment for confounders, overall the quality was fair. Nevertheless, the review points out the importance of clinical assessment as the cytokine response in leptospirosis is still poorly understood, and published literature are highly inadequate to produce a clear picture.

## Conclusions

A wide-range of cytokines were found in the circulation during a leptospirosis infection. Especially, IL-1β, IL-2, IL-4, IL-6, IL-8, IL-10 and TNF-α levels were significantly higher in severe disease. However, prospective studies with minimal sampling bias, standard diagnosis and optimum cytokine measurement in-relation to the onset of the symptom are required to better understand the pathophysiology of cytokine response in leptospirosis.

## Supplementary information

**Additional file 1.** Datasheet of the review on cytokine response in human leptospirosis, 2019. This provides the data extracted from the review.

**Additional file 2.** Summary of NIH Quality Assessment Tool for Observational Cohort and Cross-Sectional Studies. This provides the summary of quality assessment for the selected studies.

## Data Availability

All data generated or analysed during this review are included in this published article (and its additional files).

## Abbreviations

DHF: Dengue hemorrhagic fever
MAT: Microscopic agglutination test
MeSH: Medical subjects heading
PICO: Population, intervention, comparison and outcome
PRISMA: Preferred reporting items for systematic reviews and meta-analyses
VEGF: Vascular endothelial growth factors

## References

[1] Agampodi SB, Dahanayaka NJ, Bandaranayaka AK, Perera M, Priyankara S, Weerawansa P, Matthias MA, Vinetz JM (2014). Regional differences of leptospirosis in Sri Lanka: observations from a flood-associated outbreak in 2011. PLoS Negl Trop Dis.

[2] Pappas G, Papadimitriou P, Siozopoulou V, Christou L, Akritidis N (2008). The globalization of leptospirosis: worldwide incidence trends. Int J Infect Dis.

[3] Evangelista KV, Coburn J (2010). Leptospira as an emerging pathogen: a review of its biology, pathogenesis and host immune responses. Future Microbiol.

[4] Sharp TM, Rivera García B, Pérez-Padilla J, Galloway RL, Guerra M, Ryff KR, Haberling D, Ramakrishnan S, Shadomy S, Blau D, Tomashek KM, Bower WA (2016). Early indicators of fatal leptospirosis during the 2010 epidemic in Puerto Rico. PLoS Negl Trop Dis.

[5] Guerrier G, D’Ortenzio E (2013). The Jarisch-Herxheimer reaction in leptospirosis: a systematic review. PLoS One.

[6] Thaipadungpanit J, Wuthiekanun V, Chierakul W, Smythe LD, Petkanchanapong W, Limpaiboon R, Apiwatanaporn A, Slack AT, Suputtamongkol Y, White NJ, Feil EJ, Day NPJ, Peacock SJ (2007). A dominant clone of Leptospira interrogans associated with an outbreak of human leptospirosis in Thailand. PLoS Negl Trop Dis.

[7] Ganoza CA, Matthias MA, Collins-Richards D, Brouwer KC, Cunningham CB, Segura ER, Gilman RH, Gotuzzo E, Vinetz JM (2006). Determining risk for severe leptospirosis by molecular analysis of environmental surface waters for pathogenic Leptospira. PLoS Med.

[8] Wagenaar JFP, Goris MGA, Gasem MH, Isbandrio B, Moalli F, Mantovani A, Boer KR, Hartskeerl RA, Garlanda C, van Gorp ECM (2009). Long pentraxin PTX3 is associated with mortality and disease severity in severe leptospirosis. J Inf Secur.

[9] Dorigatti F, Brunialti MKC, Romero EC, Kallas EG, Salomão R (2005). *Leptospira interrogans* activation of peripheral blood monocyte glycolipoprotein demonstrated in whole blood by the release of IL-6. Brazilian J Med Biol Res = Rev Bras Pesqui medicas e Biol.

[10] Cagliero J, Villanueva SYAM, Matsui M (2018). Leptospirosis pathophysiology: into the storm of cytokines. Front Cell Infect Microbiol.

[11] Tisoncik JR, Korth MJ, Simmons CP, Farrar J, Martin TR, Katze MG (2012). Into the eye of the cytokine storm. Microbiol Mol Biol Rev.

[12] Srikiatkhachorn A, Mathew A, Rothman AL (2017). Immune-mediated cytokine storm and its role in severe dengue. Semin Immunopathol.

[13] Falasca L, Agrati C, Petrosillo N, Di Caro A, Capobianchi MR, Ippolito G, Piacentini M (2015). Molecular mechanisms of Ebola virus pathogenesis: focus on cell death. Cell Death Differ.

[14] Dickson K, Lehmann C. Inflammatory Response to Different Toxins in Experimental Sepsis Models. Int J Mol Sci. 2019;20(18):4341. 10.3390/ijms20184341 PMC6770119 31491842

[15] Czaja AJ (2014). Hepatic inflammation and progressive liver fibrosis in chronic liver disease. World J Gastroenterol.

[16] Higgins JP, Green S (2008). Editors. Cochrane handbook for systematic reviews of interventions.

[17] Moher D, Liberati A, Tetzlaff J, Altman DG (2009). PRISMA group. Preferred reporting items for systematic reviews and meta-analyses: the PRISMA statement. Ann Intern Med.

[18] NIH Quality Assessment Tool for Observational Cohort and Cross-Sectional Studies (2019). NIH National Heart, Lung and Blood Institute.

[19] Introducing Prism 8 [Internet]. GraphPad software. 2019 [cited 2019 Mar 29]. Available from: https://www.graphpad.com/scientific-software/prism/.

[20] Bandara K, Gunasekara C, Weerasekera M, Marasinghe C, Ranasinghe N, Fernando N (2018). Do the Th17 cells play a role in the pathogenesis of leptospirosis?. Can J Infect Dis Med Microbiol.

[21] Fernando N, de Silva R, Handunnetti SM, Karunanayake L, De Silva NL, de Silva HJ, Rajapakse S, Premawansa S (2018). Effect of antimicrobial agents on inflammatory cytokines in acute leptospirosis. Antimicrob Agents Chemother.

[22] Wang H, Wu Y, Ojcius DM, Yang XF, Zhang C, Ding S, Lin X, Yan J (2012). Leptospiral Hemolysins induce Proinflammatory cytokines through toll-like receptor 2-and 4-mediated JNK and NF-κB signaling pathways. PLoS One.

[23] Rizvi M, Azam M, Ajmal MR, Shukla I, Malik A (2011). Prevalence of leptospira in acute hepatitis syndrome and assessment of IL-8 and TNF-alpha level in leptospiral hepatitis. Ann Trop Med Parasitol.

[24] Kyriakidis I, Samara P, Papa A (2011). Serum TNF-α, sTNFR1, IL-6, IL-8 and IL-10 levels in Weil’s syndrome. Cytokine..

[25] Tuero I, Vinetz JM, Klimpel GR (2010). Lack of demonstrable memory T cell responses in humans who have spontaneously recovered from leptospirosis in the Peruvian Amazon. J Infect Dis.

[26] Wagenaar JFP, Gasem MH, Goris MGA, Leeflang M, Hartskeerl RA, van der Poll T, van’t Veer C, van Gorp ECM (2009). Soluble ST2 Levels Are Associated with Bleeding in Patients with Severe Leptospirosis. PLoS Negl Trop Dis.

[27] De Fost M, Chierakul W, Limpaiboon R, Dondorp A, White NJ, Van Der Poll T (2007). Release of granzymes and chemokines in Thai patients with leptospirosis. Clin Microbiol Infect.

[28] Chierakul W, de Fost M, Suputtamongkol Y, Limpaiboon R, Dondorp A, White NJ, van der Poll T (2004). Differential expression of interferon-γ and interferon-γ-inducing cytokines in Thai patients with scrub typhus or leptospirosis. Clin Immunol.

[29] Petros S, Ute LL (2000). Serum Procalcitonin and Proinflammatory cytokines in a patient with acute severe leptospirosis. Scand J Infect Dis.

[30] Cleto SA, Rodrigues CE, Malaque CM, Sztajnbok J, Seguro AC, Andrade L (2016). Hemodiafiltration decreases serum levels of inflammatory mediators in severe leptospirosis: a prospective study. PLoS One.

[31] Chirathaworn C, Supputtamongkol Y, Lertmaharit S, Poovorawan Y (2016). Cytokine levels as biomarkers for leptospirosis patients. Cytokine..

[32] Mikulski M, Boisier P, Lacassin F, Soupé-Gilbert ME, Mauron C, Bruyere-Ostells L, Bonte D, Barguil Y, Gourinat AC, Matsui M, Vernel-Pauillac F, Goarant C (2015). Severity markers in severe leptospirosis: a cohort study. Eur J Clin Microbiol Infect Dis.

[33] Papa A, Kotrotsiou T (2015). Cytokines in human leptospirosis. Trans R Soc Trop Med Hyg.

[34] Latha MP, Kaur I, Avasthi R, Dey A, Chaudhry R (2015). Cytokine profile of patient’s sera of leptospirosis to OMP of Leptospira interrogans serovar tarassovi. Asian J Med Sci.

[35] Volz MS, Moos V, Allers K, Luge E, Mayer-Scholl A, Nöckler K, Loddenkemper C, Jansen A, Schneider T (2015). Specific CD4 T-cell reactivity and cytokine release in different clinical presentations of leptospirosis. Clin Vaccine Immunol.

[36] Rizvi M, Azam M, Sultan A, Khan F, Shukla I, Malik A, Masihur R (2014). Role of IL-8 , IL-10 and TNF- α level in pathogenesis of leptospiral acute hepatitis syndrome. Ann Pathol Lab Med.

[37] Reis EAG, Hagan JE, Ribeiro GS, Teixeira-Carvalho A, Martins-Filho OA, Montgomery RR, Shaw AC, Ko AI, Reis MG (2013). Cytokine response signatures in disease progression and development of severe clinical outcomes for leptospirosis. PLoS Negl Trop Dis.

[38] Ferreira RAX, de Oliveira SA, Gandini M, Lda Ferreira C, Correa G, Abiraude FM, Reid MM, Cruz OG, Kubelka CF (2015). Circulating cytokines and chemokines associated with plasma leakage and hepatic dysfunction in Brazilian children with dengue fever. Acta Trop.

[39] Chen L-C, Lei H-Y, Liu C-C, Shiesh S-C, Chen S-H, Liu H-S, Lin Y-S, Wang S-T, Shyu H-W, Yeh T-M (2006). Correlation of serum levels of macrophage migration inhibitory factor with disease severity and clinical outcome in dengue patients. Am J Trop Med Hyg.

[40] Braga EL, Moura P, Pinto LM, Ignácio SR, Oliveira MJ, Cordeiro MT, Kubelka CF (2001). Detection of circulant tumor necrosis factor-alpha, soluble tumor necrosis factor p75 and interferon-gamma in Brazilian patients with dengue fever and dengue hemorrhagic fever. Mem Inst Oswaldo Cruz.

[41] Azeredo EL, De Oliveira-Pinto LM, Zagne SM, Cerqueira DIS, Nogueira RMR, Kubelka CF (2006). NK cells, displaying early activation, cytotoxicity and adhesion molecules, are associated with mild dengue disease. Clin Exp Immunol.

[42] Reddy V, Mani RS, Desai A, Ravi V (2014). Correlation of plasma viral loads and presence of Chikungunya IgM antibodies with cytokine/chemokine levels during acute Chikungunya virus infection. J Med Virol.

[43] Teng T-S, Kam Y-W, Lee B, Hapuarachchi HC, Wimal A, Ng L-C, Ng LFP (2015). A systematic meta-analysis of immune signatures in patients with acute Chikungunya virus infection. J Infect Dis.

[44] Chow A, Her Z, Ong EKS, Chen J, Dimatatac F, Kwek DJC, Barkham T, Yang H, Rénia L, Leo Y-S, Ng LFP (2011). Persistent arthralgia induced by Chikungunya virus infection is associated with Interleukin-6 and granulocyte macrophage Colony-stimulating factor. J Infect Dis.

[45] Morzunov SP, Khaiboullina SF, St Jeor S, Rizvanov AA, Lombardi VC (2015). Multiplex analysis of serum cytokines in humans with hantavirus pulmonary syndrome. Front Immunol.

[46] Angulo I, Fresno M (2002). Cytokines in the pathogenesis of and protection against malaria. Clin Vaccine Immunol.

[47] McElroy AK, Erickson BR, Flietstra TD, Rollin PE, Nichol ST, Towner JS, Spiropoulou CF (2014). Ebola hemorrhagic fever: novel biomarker correlates of clinical outcome. J Infect Dis.

[48] Wauquier N, Becquart P, Padilla C, Baize S, Leroy EM. Human fatal zaire ebola virus infection is associated with an aberrant innate immunity and with massive lymphocyte apoptosis. PLoS Negl Trop Dis. 2010;4(10):e837. 10.1371/journal.pntd.0000837 PMC2950153 20957152

