# Supplementary material for: Cytokine response in human leptospirosis with different clinical outcomes: a systematic review
Source: BMC Infect Dis. 2020 Apr 7;20:268. doi: 10.1186/s12879-020-04986-9 (PMC7137275; doi:10.1186/s12879-020-04986-9)
Supplement: Supplementary file 2 — Additional file 2. Summary of NIH Quality Assessment Tool for Observational Cohort and Cross-Sectional Studies. This provides the summary of quality assessment for the selected studies. [file 12879_2020_4986_MOESM2_ESM.docx]

| **Additional file 2 - Summary of Scores for NIH Quality Assessment Tool for Observational Cohort and Cross-Sectional Studies** | | | | | | | | | | | | | | | | | | | |
| --- | --- | --- | --- | --- | --- | --- | --- | --- | --- | --- | --- | --- | --- | --- | --- | --- | --- | --- | --- |
| **No** | **Criteria** | **First authors of the selected articles** | | | | | | | | | | | | | | | | | |
|  |  | Bandara K | Fernando N | Chirathaworn C | Cleto SA | Latha MP | Volz MS | Mikulski M | Papa A | Rizvi M (2014) | Reis EAG | Wang H | Rizvi M (2011) | Kyriakidis I | Tuero I | Wagenaar JFP | De Fost M | Chierakul W | Petros S |
| 1 | Was the research question or objective in this paper clearly stated? | Y | Y | Y | Y | Y | Y | Y | Y | Y | Y | Y | Y | Y | Y | Y | Y | Y | Y |
| 2 | Was the study population clearly specified and defined? | Y | Y | Y | Y | Y | Y | Y | Y | Y | Y | Y | Y | Y | Y | Y | Y | Y | NA |
| 3 | Was the participation rate of eligible persons at least 50%? | NA | NA | NA | NA | NA | NA | NA | NA | NA | NA | NA | NA | NA | NA | NA | NA | NA | NA |
| 4 | Were all the subjects selected or recruited from the same or similar populations (including the same time period)? Were inclusion and exclusion criteria for being in the study prespecified and applied uniformly to all participants? | Y | Y | Y | Y | Y | Y | Y | Y | Y | Y | Y | Y | Y | Y | Y | Y | Y | NA |
| 5 | Was a sample size justification, power description, or variance and effect estimates provided? | NR | NR | NR | NR | NR | NR | NR | NR | NR | NR | NR | NR | NR | NR | NR | NR | NR | NR |
| 6 | For the analyses in this paper, were the exposure(s) of interest measured prior to the outcome(s) being measured? | Y | Y | Y | Y | Y | Y | Y | Y | Y | Y | Y | Y | Y | Y | Y | Y | Y | Y |
| 7 | Was the timeframe sufficient so that one could reasonably expect to see an association between exposure and outcome if it existed? | Y | Y | Y | Y | Y | Y | Y | Y | Y | Y | Y | Y | Y | Y | Y | Y | Y | Y |
| 8 | For exposures that can vary in amount or level, did the study examine different levels of the exposure as related to the outcome (e.g., categories of exposure, or exposure measured as continuous variable)? | NA | NA | NA | NA | NA | NA | NA | NA | NA | NA | NA | NA | NA | NA | NA | NA | NA | NA |
| 9 | Were the exposure measures (independent variables) clearly defined, valid, reliable, and implemented consistently across all study participants? | Y | Y | Y | Y | Y | Y | Y | Y | Y | Y | Y | Y | Y | Y | Y | Y | Y | NA |
| 10 | Was the exposure(s) assessed more than once over time? | NA | NA | NA | NA | NA | NA | NA | NA | NA | NA | NA | NA | NA | NA | NA | NA | NA | NA |
| 11 | Were the outcome measures (dependent variables) clearly defined, valid, reliable, and implemented consistently across all study participants? | Y | Y | Y | Y | Y | Y | Y | Y | Y | Y | Y | Y | Y | Y | Y | Y | Y | NA |
| 12 | Were the outcome assessors blinded to the exposure status of participants? | NR | NR | NR | NR | NR | NR | NR | NR | NR | NR | NR | NR | NR | NR | NR | NR | NR | NR |
| 13 | Was loss to follow-up after baseline 20% or less? | NA | NA | NA | NA | NA | NA | NA | NA | NA | NA | NA | NA | NA | NA | NA | NA | NA | NA |
| 14 | Were key potential confounding variables measured and adjusted statistically for their impact on the relationship between exposure(s) and outcome(s)? | NR | NR | NR | NR | NR | NR | NR | NR | NR | NR | NR | NR | NR | NR | NR | NR | NR | NR |

Y - Yes, N - No, CD - Cannot Determine, NA - Not Applicable, NR - Not Reported
